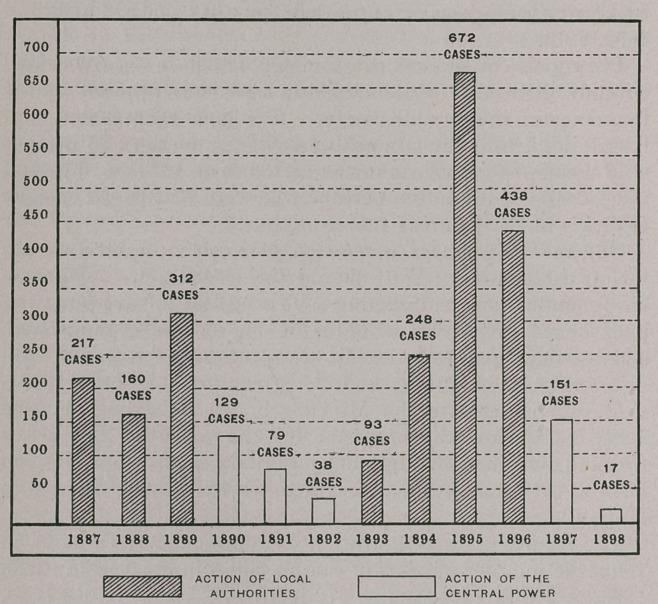# Rabies in England1From the Revue Vétérinaire, June 1, 1899.

**Published:** 1899-08

**Authors:** M. E. Leclainche

**Affiliations:** Professor of the Veterinary School of Toulouse, France


### SELECTIONS.

### RABIES IN ENGLAND.[1]

By M. E. Leclainche,
PROFESSOR OF THE VETERINARY SCHOOL OF TOULOUSE, FRANCE.

Translated by Mazÿck P. Ravenel, M.D.,
OF PHILADELPHIA.

(*From the Laboratory of the State Live-Stock Sanitary Board.*)

The history of the development of rabies during late years is interesting, for many reasons.

The act of 1878, relating to contagious diseases in animals, omits to mention the dog among those animals which require the application of sanitary measures, and until 1886 the only measures taken against rabies in the dog were imposed by the "Dogs Act" of 1871. This authorized the imposition of the muzzle in the districts where rabies had been shown to exist with certainty. The powers given to local authorities were insufficient, and, in fact, the legal prescriptions were only applied exceptionally.

The "Contagious Diseases (Animals) Act" of 1886 classes rabies of the dog among those diseases which come under the head of the general provisions of the act of 1878. By this act the Privy Council is given from henceforth the right to order the application of the muzzle in all parts of Great Britain, and to authorize the authorities of counties and boroughs to prescribe the same measure. Up to this time we have no statistical documents as to the frequency of rabies, but we know at least that the disease raged in London and in the greater part of the counties, assuming at times an epidemic character which was most alarming.

In 1887 the Privy Council decided to leave to the authorities of the counties and boroughs the charge of the sanitary police with regard to rabies. It conferred on them sufficient powers to take efficacious measures, and especially to insist on the wearing of the muzzle. At once the results were evident. From 217, in 1887, the number of cases of rabies fell to 160 in 1888, but the amelioration was only temporary, and 312 mad dogs were noted in 1889. The Metropolitan District of London alone furnished 176 cases of rabies. The Privy

[1] From the Revue Vétérinaire, June 1, 1899.

Council was aroused; it did not hesitate to substitute its authority for that of the local authorities, and it ordered the general muzzling of dogs in London within a radius of fifteen miles around Charing Cross. The result of this vigorous measure did not have to be waited for long, for in the capital there were only 44 cases of rabies seen in 1890, 28 in 1891, and 3 in 1892.

In the autumn of 1889 the office of Minister of Agriculture was created, and he inherited, in all that concerned the sanitary police of animals, the powers of the Privy Council. The success of the intervention of the central power in London was so demonstrative that the work commenced by them was continued; the muzzling was progressively imposed by the Minister in the centres most gravely invaded (Lancashire, West Riding of Yorkshire, and Cheshire). The results of this intervention are eloquently expressed by the following figures: The number of cases of rabies fell from 312 in 1889 to 129 in 1890, 79 in 1891, 38 in 1892.

During all this period the sanitary demands were accepted without resistance. Public opinion had been aroused by the frequency of the terrible disease. The journals reported with horrifying details the circumstances of the death of 25 persons who succumbed to rabies in the period from 1889 to 1892, and the journey of 147 bitten persons who were sent to the Pasteur Institute in Paris during the same time.

But, the danger over, a reaction took place; rabies was no longer threatening. Why should the wearing of the muzzle be demanded longer, inflicting such a useless torture upon the poor dogs? Numbers of clubs of dog-owners organized an active campaign; societies for the protection of animals fell into line; public opinion and the great press, its humble servant, did not sustain the Minister in his resistance, and the latter had the weakness to give in; the muzzling order was revoked, and the carrying out of sanitary measures was given back to the local authorities, which, we well know, decided to do nothing.

The results of this abdication were immediate; from 38, in 1892, the number of cases of canine rabies jumped from 93, in 1893, to 248 in 1894, and to 672 in 1895.

This last figure (672) is the highest which has been attained during the last ten years. The appalling accidents were reproduced, public opinion broke out anew, and it was necessary to put an end to rabies. In 1896 a commission was charged with the revision of all legislation relating to the question and with the preparation of a project permitting decisive action. Under the pressure of public sentiment the local authorities, moreover, aroused themselves from their inertia, and certain measures which were imposed brought as a consequence a reduction in the number of cases of rabies in the dog to 438 in 1896.

On March 23, 1897, the "Rabies Order" was promulgated. The powers conferred on the local authorities in 1886 were taken from them and given to the Minister of Agriculture. From this time on the muzzling was imposed by special orders in all infected districts, the application of the measure was gradually extended, looking to the total extinction of the disease. The inspectors of the Minister were charged with the finding of those animals bitten or exposed to contagion, requiring their slaughter or quarantine.

The results of this action are shown by statistics which need no commentary. The number of cases of rabies in the dog fell from 672 and 438 in 1895 and 1896, respectively, to 151 in

1897 and 17 in 1898. The preceding diagram brings out the only point which I wish to lay stress upon in this article, namely, the influence of decentralization in matters of sanitary police.

The collection of English official reports is filled with documents of great interest, which enable one to analyze the multiple reasons for the radical inefficiency of local authorities in the sanitary police of the contagious diseases of animals. One recalls the history, so highly suggestive, of the fight against pleuro-pneumonia in Great Britain; the history of rabies is not less demonstrative.

The English Minister has many times declared himself to be firmly resolved to fight against rabies until it shall have been totally extinguished. Already measures have been taken to prevent the reimportation of the disease. The "Dogs Order" of May 7, 1897, prohibits the importation of dogs into Great Britain unless specially authorized by the Minister of Agriculture.

The success of the campaign which has been started is not doubtful if the authorities are given a free hand for some time to come, and in a few years rabies will have disappeared from England.[1] About the same time France will exceed the annual figûre, in round numbers, of 3000 rabid dogs, holding, with Russia, the record for rabies among all nations, savage or civilized, of the two hemispheres.

The above article establishes the views of those who claim: 1st. What can be done in the prevention of rabies by proper laws; and, 2d, the importance of having such laws enforced by a central authority which takes charge of all outbreaks, decides when quarantine measures may be abolished, etc.

---

### PARASITIC DISEASE OF RABBITS.

By Veterinary Surgeon Desmond,

BACTERIOLOGIST, MELBOURNE VETERINARY COLLEGE.

From the Warrnambool district specimens of rabbits' livers that were thought to be affected with tuberculosis, or consumption, have been forwarded to me for examination.

[1] The movement of opinion which twice already has caused the work of repression to fail when almost achieved is manifesting itself at the present moment with extreme violence. In London a petition demanding the abolition of muzzling has been covered with 50,000 signatures. Will the Government once again capitulate before this popular folly?

## Figures and Tables

**Figure f1:**